# Asymptomatic Early-Stage Encapsulating Peritoneal Sclerosis Identified Laparoscopically

**DOI:** 10.1016/j.jpedcp.2024.200110

**Published:** 2024-03-28

**Authors:** Yoko Shirai, Kenichiro Miura, Taro Ando, Kazuho Honda, Osamu Segawa, Motoshi Hattori

**Affiliations:** Department of Pediatric Nephrology, Tokyo Women’s Medical University, Tokyo, Japan; Department of Anatomy, School of Medicine, Showa University, Tokyo, Japan; Department of Pediatric Surgery, Tokyo Women’s Medical University, Tokyo, Japan; Department of Pediatric Nephrology, Tokyo Women’s Medical University, Tokyo, Japan

Peritoneal dialysis (PD) is an essential treatment modality for children of any age with end-stage kidney disease, including neonates and young infants.^1^ When kidney transplantation cannot be performed for reasons such as critical inoperable cardiac disease,^2^ long-term PD is required. However, this is associated with the risk of developing encapsulating peritoneal sclerosis (EPS), a life-threatening complication characterized by abdominal pain, ultrafiltration failure, and small bowel dysfunction, which can progress to complete intestinal obstruction.^3^ Kawanishi et al reported that the incidence and mortality rates of EPS were 2.1% and 8.3% in patients undergoing PD for 8 years, respectively, and 5.9% and 28.6% in those undergoing PD for 10 years, respectively.^4^ The causes of EPS are multifactorial and include infectious peritonitis and the long-term use of bioincompatible PD fluid, in particular conventional acidic fluid, but also neutral-pH fluid with a high glucose concentration.^5^, ^6^, ^7^ Since neutral-pH PD fluid was introduced in 2000 in Japan, the incidence and clinical severity of EPS has decreased^8^; however, these complications remain a challenge in the management of PD because the diagnosis may be difficult and therefore often is delayed.^3^ Here, we report a patient who was diagnosed with very early-stage EPS during a laparoscopic procedure, despite having no compatible clinical signs.^3^

A boy with a univentricular heart underwent Fontan surgery during his infancy, but his kidney function subsequently declined as the result of bilateral kidney hypoplasia, and therefore PD was initiated at the age of 12 years. Automated PD with daytime dwell was performed using neutral-pH fluid containing 1.5%-2.5% glucose. PD was continued for 9 years because hemodialysis and kidney transplantation could not be performed, owing to arrhythmia associated with his heart disease. He experienced 2 episodes of PD-related peritonitis but had no symptoms of EPS, such as vomiting, abdominal pain, or ultrafiltration failure. He had no episode of previous abdominal surgeries except for catheter insertion. Peritoneal equilibration testing showed no increase in peritoneal permeability, and computed tomography imaging revealed no peritoneal enhancement, thickening, or calcification. Examination of a parietal peritoneum biopsy showed no evidence of peritoneal fibrosis or vasculopathy.^6^^,^^7^ He presented with refractory exit-site infection, which necessitated the simultaneous removal and reinsertion of PD catheters. Unexpectedly, laparoscopic surgery showed focal fibrotic encapsulation of his small intestine in the lower abdominal cavity, which was compatible with a very early stage of EPS (Figure). There were no alternate causes for fibrosis or adhesion of the peritoneum. Corticosteroid therapy was initiated, because it has been described to be potentially effective for EPS.^9^ In addition, the patient is preparing for pacemaker implantation and a switch from PD to hemodialysis. Informed consent was obtained from the patient and his guardian.

**Figure:**
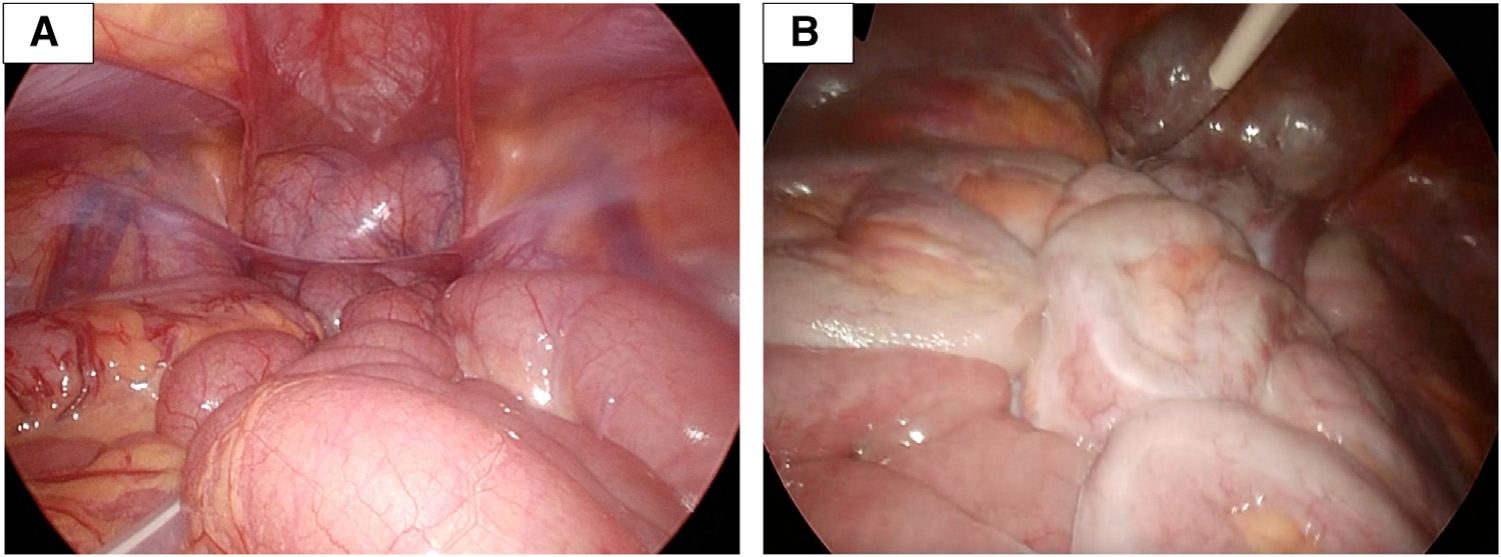
Laparoscopic findings of the peritoneum. **A,** Normal peritoneal image of a control patient with end-stage kidney disease who underwent PD catheter insertion. **B,** Focal fibrotic encapsulation of the small intestine was identified during laparoscopic surgery.

This case suggests that despite the use of biocompatible dialysate, EPS can progress in the absence of either ultrafiltration failure or pathologic changes associated with peritoneal sclerosis. Laparoscopic examination may be a useful means of identifying early-stage EPS, contributing to adequate management of patients undergoing long-term PD for more than 8 years.

## CRediT authorship contribution statement

**Yoko Shirai:** Writing – original draft, Investigation, Data curation, Conceptualization. **Kenichiro Miura:** Writing – review & editing, Investigation, Funding acquisition. **Taro Ando:** Data curation. **Kazuho Honda:** Writing – review & editing, Supervision, Investigation, Conceptualization. **Osamu Segawa:** Investigation, Data curation. **Motoshi Hattori:** Writing – review & editing, Supervision, Investigation, Conceptualization.

## Declaration of Competing Interest

This work was supported by a Grant-in-Aid for Intractable Renal Diseases Research, Research on Rare and Intractable Diseases, Health and Labour Sciences Research Grants from the Japan Ministry of Health, Labour, and Welfare and Medical Research Institute (MRI), 10.13039/501100010391Tokyo Women’s Medical University. The authors declare no conflicts of interest.
